# Genome-wide association study of metabolic dysfunction-associated fatty liver disease in a Korean population

**DOI:** 10.1038/s41598-024-60152-0

**Published:** 2024-04-29

**Authors:** Young Lee, Eun Ju Cho, Eun Kyung Choe, Min-Sun Kwak, Jong In Yang, Seung-Won Oh, Jeong Yoon Yim, Goh Eun Chung

**Affiliations:** 1 Veterans Medical Research Institute, Veterans Health Service Medical Center, Seoul, Republic of Korea; 2 Department of Applied Statistics, Chung-Ang University, Seoul, Republic of Korea; 3 Department of Internal Medicine and Liver Research Institute, Seoul National University College of Medicine, Seoul, Republic of Korea; 4 Department of Healthcare Research Institute, Seoul National University Hospital Healthcare System Gangnam Center, Seoul, Republic of Korea; 5 Department of Family Medicine, Seoul National University Hospital Healthcare System Gangnam Center, Seoul National University College of Medicine, Seoul, Republic of Korea

**Keywords:** Metabolic fatty liver disease, Genome-wide association study, SNP, Hepatology, Liver

## Abstract

Genome-wide association studies have identified several genetic variants associated with nonalcoholic fatty liver disease. To emphasize metabolic abnormalities in fatty liver, metabolic (dysfunction)-associated fatty liver disease (MAFLD) has been introduced; thus, we aimed to investigate single-nucleotide polymorphisms related to MAFLD and its subtypes. A genome-wide association study was performed to identify genetic factors related to MAFLD. We used a Korean population-based sample of 2282 subjects with MAFLD and a control group of 4669. We replicated the results in a validation sample which included 639 patients with MAFLD and 1578 controls. Additionally, we categorized participants into three groups, no MAFLD, metabolic dysfunction (MD)-MAFLD, and overweight/obese-MAFLD. After adjusting for age, sex, and principal component scores, rs738409 [risk allele G] and rs3810622 [risk allele T], located in the *PNPLA3* gene, showed significant associations with MAFLD (*P*-values, discovery set = 1.60 × 10^–15^ and 4.84 × 10^–10^; odds ratios, 1.365 and 1.284, validation set = 1.39 × 10^–4^, and 7.15 × 10^–4^, odds ratios, 1.299 and 1.264, respectively). An additional SNP rs59148799 [risk allele G] located in the *GATAD2A* gene showed a significant association with MAFLD (*P*-values, discovery set = 2.08 × 10^–8^ and validation set = 0.034, odds ratios, 1.387 and 1.250). rs738409 was significantly associated with MAFLD subtypes ([overweight/obese-MAFLD; odds ratio (95% confidence interval),* P*-values, 1.515 (1.351–1.700), 1.43 × 10^–12^ and MD-MAFLD: 1.300 (1.191–1.416), 2.90 × 10^–9^]. There was a significant relationship between rs3810622 and overweight/obese-MAFLD and MD-MAFLD [odds ratios (95% confidence interval), *P*-values, 1.418 (1.258, 1.600), 1.21 × 10^–8^ and 1.225 (1.122, 1.340), 7.06 × 10^–6^, respectively]; the statistical significance remained in the validation set. *PNPLA3* was significantly associated with MAFLD and MAFLD subtypes in the Korean population. These results indicate that genetic factors play an important role in the pathogenesis of MAFLD.

## Introduction

As the most common cause of chronic liver disease worldwide, the global trend of non-alcoholic fatty liver disease (NAFLD) represents an important public health problem^1,2^. Both environmental and genetic factors play important roles in the development of NAFLD^3,4^. With recent advances in genetic research, genome-wide association studies (GWASs) have identified several genetic variants. The most replicated variants associated with NAFLD include patatin-like phospholipase domain-containing 3 (*PNPLA3*), transmembrane 6 superfamily member 2 (*TM6SF2*), hydroxysteroid 17-beta dehydrogenase 13 (*HSD17B13*), membrane bound O-acyltransferase domain containing 7 (*MBOAT7*), glucokinase regulator (*GCKR*) and *SAMM50*^5,6^.

A new definition of metabolic (dysfunction)-associated with fatty liver disease (MAFLD) has been introduced, emphasizing the role of metabolic dysfunction in the clinical outcomes of patients with hepatic steatosis^7,8^. MAFLD criteria have demonstrated improved capability in identifying a subset of patients with more advanced liver disease and higher mortality compared to those diagnosed solely with NAFLD^9–11^. Moreover, in MAFLD patients, MAFLD subtypes have proven to be effective in capturing differential outcomes related to cardiovascular disease or disease-specific mortality^12,13^.

Although MAFLD and NAFLD overlap considerably and both disease entities have similar clinical features^14^, utilizations of the MAFLD criteria have identified more individuals with advanced liver disease and patients at a high risk for developing complications or other metabolic co-morbidities^15,16^. Therefore, investigating genetic variants associated with MAFLD can provide insight into the pathogenesis of MAFLD and help stratify high-risk patients. A recent study reported no association between a single nucleotide polymorphism (SNP) and the risk of MAFLD in a Han population^17^; however, studies investigating the influence of genetic susceptibility on MAFLD are limited. In this study, we investigated the association between genome-wide SNP and MAFLD and its subgroups in an asymptomatic Korean population.

## Materials and methods

### Study population

We analyzed the Gene-Environment Interaction and phenotype cohort data as previously described^18^. From January 2014 and December 2014, 9676 people donated blood samples during regular health check-ups conducted at Seoul National University Hospital Healthcare System Gangnam Center. Their blood samples were stored in a biorepository with informed consent. Participants with missing data from the total enrollment were excluded. The enrolled population was divided into two groups based on the time of enrollment. Samples from subjects enrolled between January 2014 and October 2014 were used as the discovery set, and samples from subjects enrolled in subsequent months were used as the validation set. Results using the discovery set were verified using the validation set.

The Institutional Review Board (IRB) approved the storage of biospecimens with informed consent (Seoul National University Hospital/IRB number 1103-127-357). Because this study used anonymized clinical and genetic information collected and analyzed previously, the board approved this study protocol, and the informed consent was waived (Seoul National University Hospital/IRB number 2109-012-1251). This research was performed in accordance with the Declaration of Helsinki.

### Clinical and laboratory assessments

Each subject completed a medical history questionnaire and received an anthropometric evaluation. Each participant’s weight was measured using a digital scale. Body mass index (BMI) was calculated as the weight (kg) divided by height (m) squared. Waist circumference was measured at the midpoint between the lower costal margin and anterior superior iliac crest by a well-trained person. Systolic and diastolic blood pressures were measured twice, and the mean values were reported.

The laboratory and radiological tests were performed on the same day. Blood specimens were obtained from each participant after an overnight fast of ≥ 8 h. Laboratory evaluations included serum total cholesterol, triglyceride, high-density lipoprotein (HDL) cholesterol, fasting glucose, and high-sensitivity C-reactive protein. Hypertension was defined as a systolic blood pressure ≥ 140 mm Hg, diastolic blood pressure ≥ 90 mm Hg, or use of antihypertensive agents. Diabetes mellitus was defined as a fasting serum glucose level of ≥ 126 mg/dL or glycosylated hemoglobin ≥ 6.5% or the use of antidiabetic medications.

The diagnosis of fatty liver was based on ultrasonographic findings (Acusion, Sequoia 512, Siemens, Mountain View, CA, USA) of experienced radiologists who were unaware of the clinical information. Sonographic features of hepatic steatosis were classified according to previously described protocols^13,14^.

### Definition of MAFLD and subgroups

MAFLD was defined as the presence of metabolic risk factors in the setting of hepatic steatosis based on the diagnostic criteria proposed by an international consensus in 2020^7^. This definition does not exclude those with other concomitant liver diseases or significant alcohol consumption. MAFLD was diagnosed as the presence of hepatic steatosis with one or more of the following: (1) overweight or obese (BMI ≥ 23 kg/m^2^), (2) diabetes mellitus, or (3) at least two metabolic risk abnormalities. Metabolic risk abnormalities consisted of (1) waist circumference (WC) ≥ 90 cm for men and 80 ≥ cm for women, (2) blood pressure ≥ 130/85 mm Hg or specific drug treatment, (3) fasting plasma triglycerides ≥ 150 mg/dL or specific drug treatment, (4) plasma HDL-cholesterol < 40 mg/dl for men and < 50 mg/dL for women or specific drug treatment, (5) prediabetes (fasting glucose 100–125 mg/dL or hemoglobin A1c 5.7–6.4%), (6) homeostasis model assessment of insulin resistance score ≥ 2.5, (7) plasma high-sensitivity C-reactive protein level > 2 mg/dL. As a homeostasis model assessment of the insulin resistance score was not available, this criterion was not implemented in our study.

In the present study, participants with MAFLD were classified into two groups, modified from a previous study^19^. First, we determined MAFLD-metabolic dysfunction (MD) subgroup classification based on the presence of diabetes or at least two metabolic abnormalities regardless of the BMI; the remaining non-diabetic subjects who had fewer than two metabolic abnormalities and met only the BMI ≥ 23 kg/m^2^ criteria of MAFLD were categorized as the MAFLD-overweight/obese group.

### Genotyping and quality control

Genomic DNA was extracted from blood samples and 200 ng of DNA from each patient was genotyped using Affymetrix Axiom® Customized Biobank Genotyping Arrays (Affymetrix, Santa Clara, CA, USA). Genotype data were produced using the Korean Chip, designed by the Center for Genome Science, Korea National Institute of Health, Korea (4845–301, 3000–3031). The quality control measures were performed using the PLINK program (version 1.07; Free Software Foundation Inc., Boston, MA, USA). Samples meeting any of the following criteria were removed: (i) gender discrepancy, (ii) call rate ≤ 97%, and (iii) related and cryptically related individuals (identical by state > 90%). SNPs were filtered if (1) the call rate was < 95%, (2) minor allele frequency was ≤ 0.05, or (3) deviation from the Hardy–Weinberg equilibrium permutation test (*P* < 1.00 × 10^−5^) was observed. SHAPEIT2 v2.r904 and IMPUTE2 version 2.3.2 were used for pre-phasing the data and genotype imputations in GWAS.^20,21^ As a reference panel, we used 1000 Genomes Phase 3 haplotypes. Any imputed SNPs with imputation quality scores < 0.5 were excluded. After quality control, 3,631,724 SNPs in the discovery set and 3,587,584 SNPs in the validation set remained for association analysis.

### Statistical analysis

Comparisons of continuous variables in the baseline characteristics between the two groups (discovery set vs validation set and no MAFLD vs. MAFLD) were performed using Student’s *t*-test, and categorical variables were compared using the chi-square test or Fisher’s exact test. Binary logistic regression analysis was used to analyze the influence of SNPs on MAFLD. Ordinal and multinomial logistic regression analyses were performed to investigate the association of SNPs among MAFLD subgroups. The analysis was performed using an additive genetic model, adjusting for age, sex, and five principal component scores. The genomic inflation factor (*λ*) was computed to confirm that population stratification did not cause confounding. A *λ* close to 1 indicates the absence of genomic inflation^22^. SNPs with a *P-*value of less than 5.0 × 10^–8^, which is conventionally considered to represent genome-wide significance, in the discovery set were re-evaluated in the validation set. We also performed a joint analysis on the combined samples. To evaluate the independence of association signals, we also performed a conditional analysis.

The PLINK software package (version 1.07), R statistical software package (version 3.1.1, R Development Core Team; R Foundation for Statistical Computing, Vienna, Austria), and Trinculo (Bayesian and frequentist multinomial logistic regression for GWASs of multicategory phenotypes) were used to test the association between MAFLD and SNPs in the genome and draw a Manhattan plot and quantile–quantile (Q-Q) plots. We used LocusZoom and linkage disequilibrium data from East Asians in the 1000 Genomes Project to generate a regional plot of significant genetic variation^23^.

## Results

### Study population

A total of 9168 subjects were included, of whom 58.6% were male. Based on the definitions described in the Methods section, there were 6951 samples in the discovery set and 2217 samples in the validation set. Characteristics of the study population are summarized in Table 1. The subjects in the validation set were older and had higher BMI, WC, and fasting glucose and lower proportions of MAFLD (*P* < 0.05). Most anthropometric and laboratory variables were less metabolically favorable in subjects with MAFLD than those without MAFLD (all *P* < 0.001, Table 1).

**Table 1:** Baseline characteristics of the study population.

	Discovery set (*n* = 6951)	Validation set (*n* = 2217)	*P*-value	No MAFLD (*n* = 6247)	MAFLD (*n* = 2921)	*P*-value
Age (years)	50.5 ± 10.0	51.1 ± 9.8	0.031	50.0 ± 10.2	52.1 ± 9.2	< 0.001
Male,* n* (%)	4036 (58.1)	1336 (60.3)	0.067	3059 (49.0)	2313 (79.2)	< 0.001
Diabetes mellitus,* n* (%)	330 (4.7)	127 (5.7)	0.065	174 (2.8)	283 (9.7)	< 0.001
Hypertension,* n* (%)	1163 (16.7)	388 (17.5)	0.400	744 (11.9)	807 (27.6)	< 0.001
Body mass index (kg/m^2^)	23.1 ± 3.0	23.6 ± 3.1	< 0.001	22.1 ± 2.6	25.6 ± 2.5	< 0.001
Waist circumference (cm)	82.5 ± 8.8	84.1 ± 9.1	< 0.001	79.7 ± 7.8	90.0 ± 6.7	< 0.001
Cholesterol (mg/dL)	191.3 ± 38.5	193.1 ± 45.6	0.094	190.6 ± 39.1	194.3 ± 42.8	< 0.001
Triglycerides (mg/dL)	108.0 ± 73.9	110.7 ± 79.9	0.160	89.9 ± 52.6	148.7 ± 97.7	< 0.001
HDL cholesterol (mg/dL)	53.9 ± 12.0	52.7 ± 11.8	< 0.001	56.1 ± 12.2	48.2 ± 9.4	< 0.001
Fasting glucose (mg/dL)	98.2 ± 16.5	101.0 ± 20.0	< 0.001	95.5 ± 14.5	106.1 ± 21.3	< 0.001
Hs-CRP (mg/dL)	0.1 ± 0.3	0.1 ± 0.3	0.810	0.1 ± 0.3	0.2 ± 0.3	< 0.001
MAFLD, *n* (%)	2282 (32.8)	639 (28.8)	< 0.001			
MAFLD subtype, *n* (%)			0.001			
No MAFLD	4669 (67.2)	1578 (71.2)				
Overweight/obese MAFLD	737 (10.6)	192 (8.7)			939 (31.8)	
MD-MAFLD	1545 (22.2)	447 (20.2)			1992 (68.2)	

Data are shown as the mean ± SD.

MAFLD, metabolic dysfunction associated fatty liver disease; HDL, high-density lipoprotein; hs-CRP, high sensitivity C-reactive protein; MD, metabolic dysfunction.

### GWAS of MAFLD

**Figure 1:**
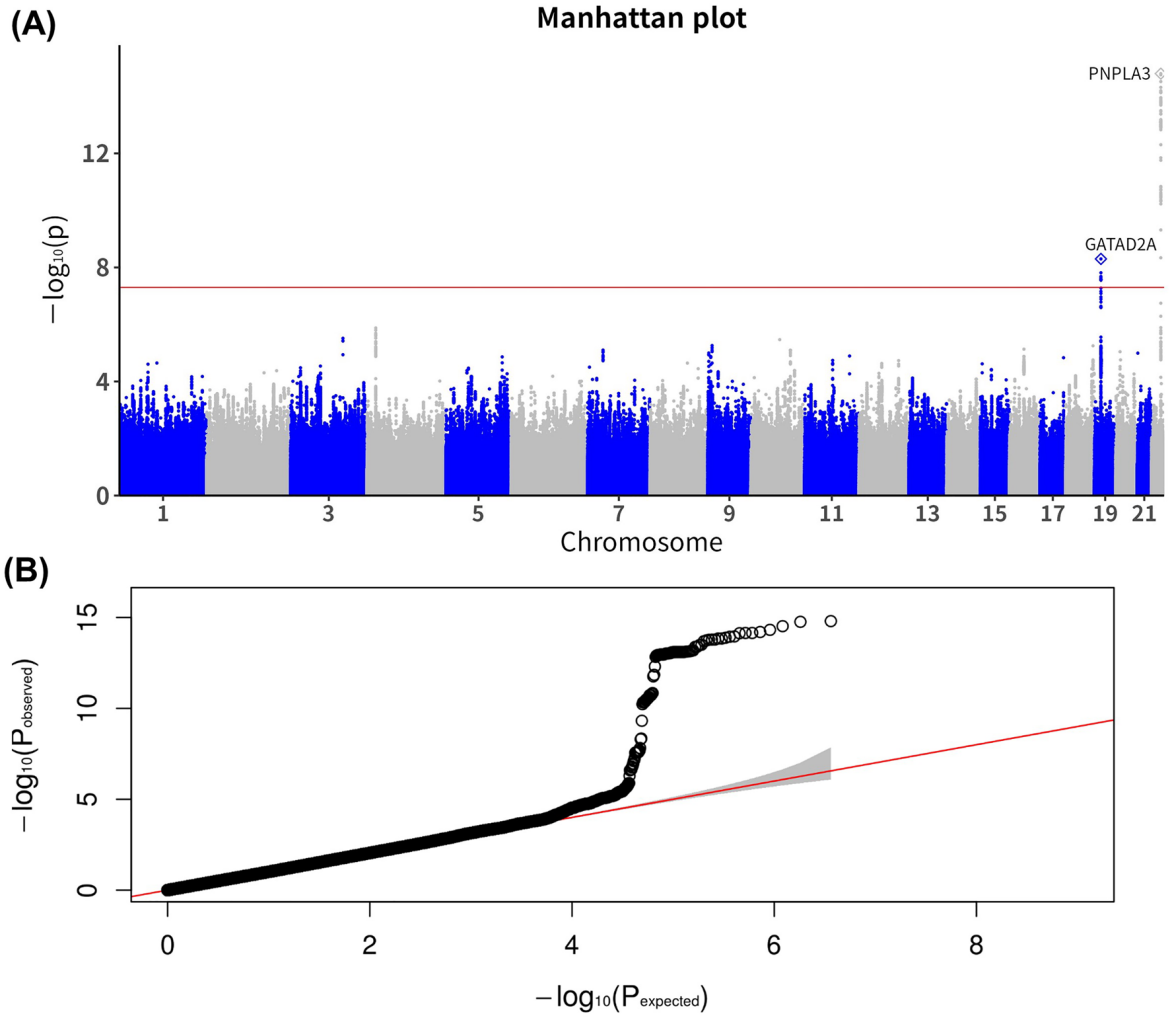
Manhattan and quantile–quantile (Q–Q) plot for single nucleotide polymorphism in the discovery set. (**A**) Manhattan plot of the *P*-values in the genome-wide association studies for MAFLD. The horizontal dash line indicates the preset threshold of *P* = 5.0 × 10^–8^. (**B**) Q–Q plot showing expected versus observed (–log_10_ *P*-value). The expected line is shown in red and confidence bands are shown in gray.

**Figure 2:**
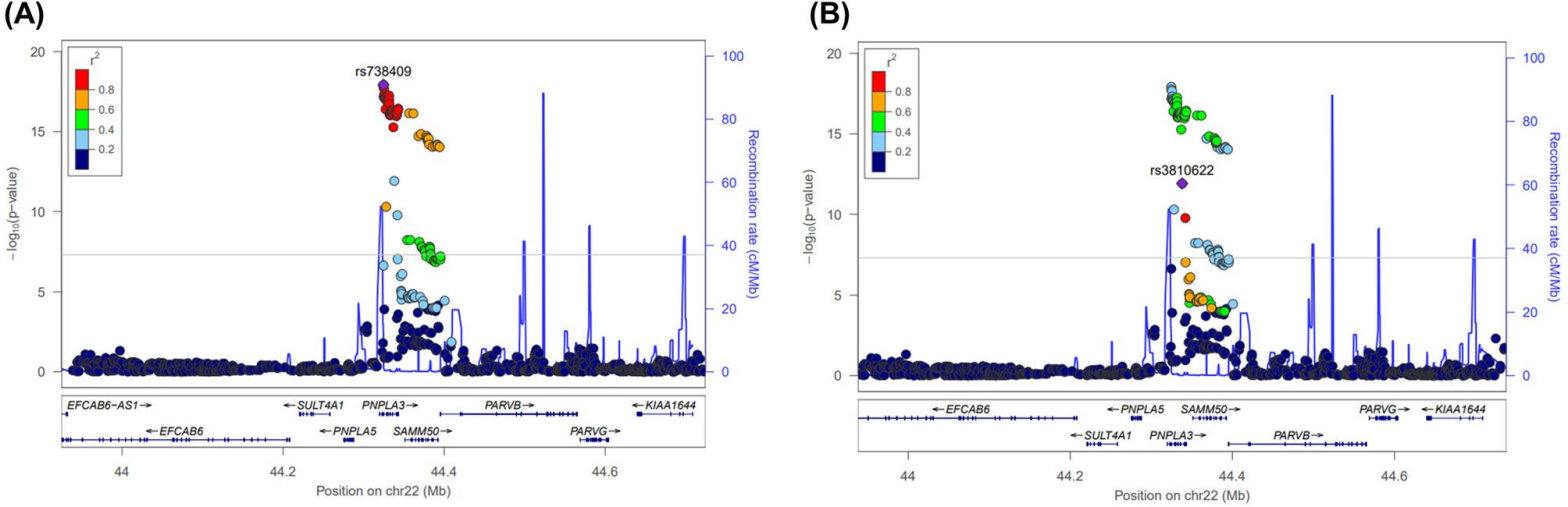
Regional association plots for chromosome 22 (**A**) rs738409 (**B**) rs3810622. Nearby SNPs are color-coded according to the level of linkage disequilibrium with the top SNP. The left y-axis represents the significance of the association based on the -log_10_ *P*-value (logistic regression in the combined samples), and the right y-axis represents the recombination rate across regions. SNP, single nucleotide polymorphism.

**Figure 3:**
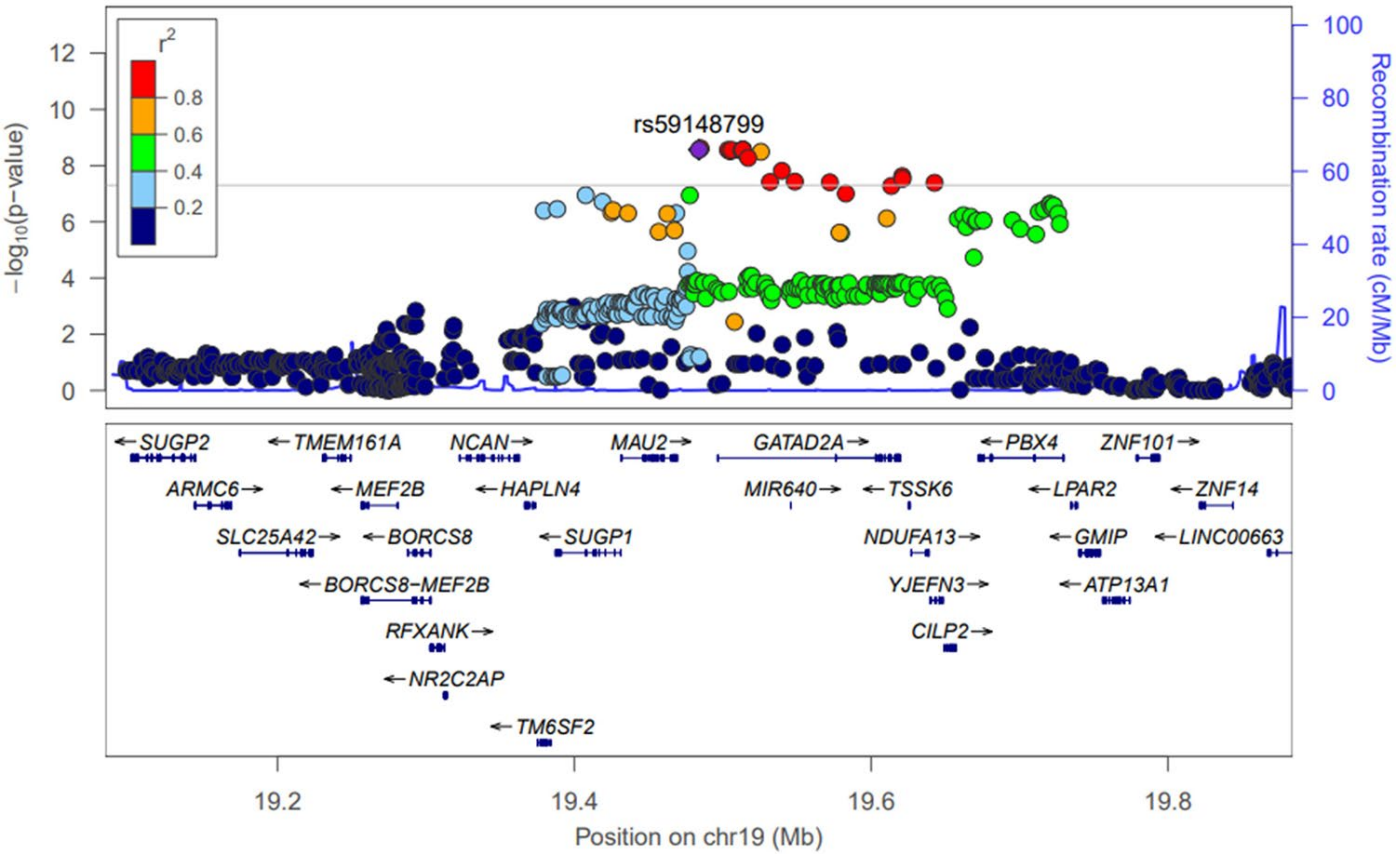
Regional association plots for chromosome 19. Nearby SNPs are color-coded according to the level of linkage disequilibrium with the top SNP. The left y-axis represents the significance of the association based on the -log_10_ *P*-value (logistic regression in the combined samples), and the right y-axis represents the recombination rate across regions. SNP, single nucleotide polymorphism.

We first evaluated the genome-wide associations of MAFLD in the discovery set, with a significance threshold of *P* < 5.0 × 10^–8^, after adjusting for age, sex, and principal component scores. Figure 1 shows the Manhattan and Q-Q plots. The Q-Q plot revealed no evidence of any inflation of the test statistics (*λ* = 1.012, min 0.997 max 1.021). The significant SNPs in the discovery set that have been successfully replicated are listed in Table 2. In the GWAS discovery set, the two top SNPs on 22 chromosomes were significantly associated with MAFLD, which remained significant in the validation set. rs738409 and rs3810622 located in the *PNPLA3* gene showed significant associations with MAFLD (*P*-values, discovery set = 1.60 × 10^–15^ and 4.84 × 10^–10^, odds ratio [OR], 1.365 and 1.284, respectively; validation set = 1.39 × 10^–4^ and 7.15 × 10^–4^, OR, 1.299 and 1.264, respectively). A regional plot of chromosome 22 is shown in Fig. 2. An additional SNP rs59148799, located in the GATA zinc finger domain containing 2A (*GATAD2A*) gene on chromosome 19, showed a significant association with MAFLD (*P*-values, discovery set = 2.08 × 10^–8^ and validation set = 0.034, Table 2). A regional plot of chromosome 19 is shown in Fig. 3. When we performed a joint analysis, these significant associations remained (Table 2).

**Table 2:** SNP associated with metabolic dysfunction associated fatty liver disease.

					Discovery		Validation		Joint	
SNP	Chr	Position	Nearest Genes	Risk Allele	OR (95% CI)	*P*-value	OR (95% CI)	*P*-value	OR (95% CI)	*P*-value
rs738409	22	44,324,727	*PNPLA3*	G	1.365 (1.264, 1.473)	1.60E-15	1.299 (1.136, 1.487)	1.39E-04	1.348 (1.261, 1.440)	1.20E-18
rs3810622	22	44,338,134	*PNPLA3*	T	1.284 (1.186, 1.389)	4.84E-10	1.264 (1.104, 1.447)	7.15E-04	1.279 (1.195, 1.369)	1.22E-12
rs59148799	19	19,484,008	*GATAD2A*	G	1.387 (1.237, 1.555)	2.08E-08	1.250 (1.017, 1.536)	0.034	1.353 (1.225, 1.495)	2.71E-09

*P*-values are calculated by binary logistic regression adjusted for age, sex, and five principal component scores assuming additive genetic model. Genomic position is based on NCBI build 37.

SNP, single nuclear polymorphism; CI, confidence interval; OR, odds ratio; Chr, chromosome.

We also performed a conditional analysis to evaluate the independence of association signals. Conditional analysis with multiple lead SNPs on same chromosome showed that the effect of rs3810622 on MAFLD disappeared, but the significance of rs738409 remained even when rs3810622 was evaluated together (Supplementary Table 1).

### Impact of SNPs on MAFLD subtypes

Next, we investigated the associations between SNPs identified in the GWAS and MAFLD subtypes (no MAFLD, overweight/obese MAFLD, and MD-MAFLD). Ordinal logistic regression analysis after adjusting for age, sex, and five principal component scores showed that two SNPs, rs738409 and rs3810622, located in the *PNPLA3* gene, showed significant associations with MAFLD subtypes (*P*-values, discovery set = 6.63 × 10^–13^, and 2.71 × 10^–8^, validation set = 5.54 × 10^–4^, and 0.002, Joint set = 1.64 × 10^–15^, and 1.75 × 10^–10^, respectively; Table 3). When we performed a conditional analysis, the independent association of rs738409 remained even when rs3810622 was evaluated together (Supplementary Table 2).

**Table 3:** SNP associated with the subtype of metabolic dysfunction associated fatty liver disease.

					Discovery		Validation		Joint	
SNP	Chr	Position	Nearest Genes	Risk Allele	OR (95% CI)	*P*-value	OR (95% CI)	*P*-value	OR (95% CI)	*P*-value
rs738409	22	44,324,727	*PNPLA3*	G	1.309 (1.216, 1.409)	6.63E-13	1.299 (1.105, 1.438)	5.54E-04	1.348 (1.217, 1.383)	1.64E-15
rs3810622	22	44,338,134	*PNPLA3*	T	1.239 (1.149, 1.335)	2.71E-08	1.231 (1.079, 1.405)	0.002	1.238 (1.159, 1.321)	1.75E-10

*P*-values are calculated by ordinal logistic regression (proportional odds model) adjusted for age, sex, and five principal component scores assuming additive genetic model.

Genomic position is based on NCBI build 37.

SNP, single nuclear polymorphism; CI, confidence interval; OR, odds ratio; Chr, chromosome.

We investigated the ORs of each SNP of the MAFLD subtype compared to the group without MAFLD. Associations between the two SNPs and MAFLD subtypes are shown in Table 4. rs738409 was associated with a higher risk of overweight/obese MAFLD and MD-MAFLD [discovery set, OR (95% confidence interval (CI)), *P*-value, 1.515 (1.351–1.700), 1.43 × 10^–12^ and 1.300 (1.191–1.416), 2.90 × 10^–9^, respectively; validation set, OR (95% CI), *P*-value, 1.466 (1.182–1.818), 0.0005 and 1.233 (1.058–1.437), 0.0073, respectively)]. Additionally, significant associations were observed between rs3810622 and overweight/obese MAFLD and MD-MAFLD [discovery set, OR (95% CI), *P*-value, 1.418 (1.258, 1.600), 1.21 × 10^–8^ and 1.225 (1.122, 1.340), 7.06 × 10^–6^; validation set, OR (95% CI), *P*-value, 1.453 (1.164, 1.815), 9.76 × 10^–4^ and 1.192 (1.022, 1.389), 0.0248, respectively]. When we performed a joint analysis, these significant associations remained (Table 4). A conditional analysis with multiple lead SNPs showed that the effect of rs738409 on MAFLD remained significant when rs3810622 was evaluated together (Supplementary Table 3).

**Table 4:** The association between SNPs and subtype of metabolic dysfunction associated fatty liver disease.

SNP	Risk Allele		Discovery		Validation		Joint	
rs738409	G	MAFLD subtype	OR (95% CI)	*P*-value	OR (95% CI)	*P*-value	OR (95% CI)	*P*-value
		No MAFLD	1 (reference)		1 (reference)		1 (reference)	
		Overweight/obese MAFLD	1.515 (1.351, 1.700)	1.43E-12	1.466 (1.182, 1.818)	0.0005	1.504 (1.359, 1.664)	3.08E-15
		MD-MAFLD	1.300 (1.191, 1.416)	2.90E-09	1.233 (1.058, 1.437)	0.0073	1.282 (1.189, 1.382)	9.34E-11

			Discovery		Validation		Joint	
rs3810622	T	MAFLD subtype	OR (95% CI)	*P*-value	OR (95% CI)	*P*-value	OR (95% CI)	*P*-value
		No MAFLD	1 (reference)		1 (reference)		1 (reference)	
		Overweight/obese MAFLD	1.418 (1.258, 1.600)	1.21E-08	1.453 (1.164, 1.815)	9.76E-04	1.426 (1.283, 1.585)	4.28E-11
		MD-MAFLD	1.225 (1.122, 1.340)	7.06E-06	1.192 (1.022, 1.389)	0.0248	1.217 (1.127, 1.314)	5.06E-07

*P*-values are calculated by multinomial logistic regression adjusted for age, sex, and five principal component scores assuming additive genetic model. Genomic position is based on NCBI build 37.

MAFLD, metabolic dysfunction associated fatty liver disease; CI, confidence interval; OR, odds ratio; MD, metabolic dysfunction.

## Discussion

Although the main pathogenesis is common between NAFLD and MAFLD, there have been no GWAS on MAFLD in Korea. In this study, we found a significant association between *PNPLA3* and *GATAD2A* gene variants and MAFLD in a Korean population using GWAS. Moreover, we demonstrated that *PNPLA3* rs738409 and rs3810622 were associated with MAFLD subtypes.

A recent review investigated the effects and applications of SNPs associated with MAFLD; however, the disease groups included in most of the studies were NAFLD rather than pure MAFLD^24^. A previous study based on the Han population found no association between SNPs and the risk of MAFLD in Western China^17^. Some significant associations may not be found because the sample size was not large enough (286 cases and 250 healthy controls). Recently, a systematic review reported genetic predispositions in MAFLD showing that rs641738C > T near membrane-bound O-acyltransferase domain-containing 7 is associated with increased hepatic fat, MAFLD severity, susceptibility to develop nonalcoholic steatohepatitis, advanced fibrosis, and hepatocellular carcinoma in Western populations with MAFLD^25^. However, these results were not based on GWAS.

In this study, three SNPs were significantly associated with MAFLD. Among them, rs738409 encodes *PNPLA3* I148M, which is associated with increased hepatic fat in multiple ethnicities^26–29^. The *PNPLA3* gene, located in hepatic lipid droplets, is involved in lipid metabolism by interfering with adipose triglyceride lipase activity and modulates hepatic triglyceride accumulation^30,31^. The *PNPLA3* rs3810622 intron variant has been previously reported to be associated with elevated alanine aminotransferase and blood glucose^32^ and decreased serum triglyceride in NAFLD patients, suggesting its role in MAFLD as well as NAFLD^33,34^.

Additionally, rs59148799, located in *GATAD2A* showed a significant association with MAFLD. Although bioinformatic analysis has shown that this SNP is an intronic polymorphism that does not influence the protein structure or RNA splicing, it remains unclear whether transcription factor-binding sites are located within this SNP. In line with our results, *GATAD2A* rs4808199 has been reported to be associated with NAFLD in Japanese^35^ and U.S. multi‐ethnic populations^36^ although MAFLD was not considered in these studies. In contrast, *GATAD2A* rs4808199 showed no significant correlation with MAFLD in Western Chinese^17,37^. Collectively, further studies are required to confirm the association between *GATAD2A* and MAFLD.

*PNPLA3* and *SAMM50* genes were known to be significantly associated with the presence and severity of NAFLD in a Korean population in a previous study^6^. Compared to a previous study in Korean population^6^, the phenotype in this study focused on MAFLD and did not exclude heavy alcohol drinkers, or patients with concomitant liver disease. *PNPLA3* variants showed a significant association with MAFLD as well as NAFLD, but no association was found between *SAMM50* variants and MAFLD in this study. Other variants in the *TM6SF2*, *HSD17B13*, *MBOAT7* and *GCKR* genes previously known to be associated with NAFLD were not found to be associated with MAFLD in this study. Ethnic differences among study populations and differences in outcome phenotypes may cause differences in results.

MAFLD affects a heterogeneous group of patients, including overweight/obese patients without metabolic abnormalities and lean patients with metabolic dysfunction. In terms of mortality, the MAFLD subgroups showed differential outcomes according to the accompanying metabolic dysfunctions^13,38^. On investigating the associations between SNPs and MAFLD subtypes, rs738409 and rs3810622, located in *PNPLA3*, showed significant associations with MAFLD subtypes. Further, there were significant relationships in both overweight/obese MAFLD and MD-MAFLD patients, suggesting possible genetic associations between *PNPLA3* gene and MAFLD subtypes.

The present study had certain limitations. First, the discovery and validation sets selected were from the same population that underwent regular health checkups at our institute. Further validation studies using other populations should be conducted. Second, although ultrasonography is widely used as a first-line method to detect hepatic steatosis in clinical practice^39,40^, it is relatively insensitive for detecting mild steatosis^41^. Third, a definition of metabolic dysfunction-associated steatotic liver disease (MASLD) has been introduced recently, which overcomes the exclusive and stigmatizing nature of the NAFLD and the limitations of MAFLD^42^. Because this study focused on MAFLD rather than NAFLD or MASLD, we did not exclude, or distinguish concomitant liver disease or significant alcohol use according to the MAFLD definition. Therefore, samples and observations may be biased with respect to NAFLD or MASLD. Additionally, we did not consider disease activity or severity in this study. Finally, there may be selection bias because the study population may not be representative of the general population of subjects who visit a single health screening center in Korea for health checkups.

In conclusion, *PNPLA3* was significantly associated with MAFLD and its subtypes in the Korean population. Our findings indicate that genetic factors play important roles in the pathogenesis of MAFLD and NAFLD.

### Supplementary Information

Supplementary Tables.

## Data Availability

The datasets used and/or analyzed during the current study are available from the corresponding author on reasonable request**.**
