# Author Correction: Genome-wide association study of metabolic dysfunction-associated fatty liver disease in a Korean population

**DOI:** 10.1038/s41598-024-61718-8

**Published:** 2024-05-14

**Authors:** Young Lee, Eun Ju Cho, Eun Kyung Choe, Min-Sun Kwak, Jong In Yang, Seung-Won Oh, Jeong Yoon Yim, Goh Eun Chung

**Affiliations:** 1 Veterans Health Service Medical Center, Veterans Medical Research Institute, Seoul, Republic of Korea; 2 Department of Applied Statistics, Chung-Ang University, Seoul, Republic of Korea; 3 Department of Internal Medicine and Liver Research Institute, Seoul National University College of Medicine, Seoul, Republic of Korea; 4 Department of Healthcare Research Institute, Seoul National University Hospital Healthcare System Gangnam Center, Seoul, Republic of Korea; 5 Department of Family Medicine, Seoul National University Hospital Healthcare System Gangnam Center, Seoul National University College of Medicine, Seoul, Republic of Korea

Correction to: *Scientific Reports* 10.1038/s41598-024-60152-0, published online 29 April 2024

The original version of this Article contained an error in affiliation 4. The correct affiliation is listed below.

Department of Healthcare Research Institute, Seoul National University Hospital Healthcare System Gangnam Center, Seoul, Republic of Korea.

In addition, an affiliation was omitted for Seung-Won Oh. The correct affiliations for Seung-Won Oh are listed below.

Department of Healthcare Research Institute, Seoul National University Hospital Healthcare System Gangnam Center, Seoul, Republic of Korea.

Department of Family Medicine, Seoul National University Hospital Healthcare System Gangnam Center, Seoul National University College of Medicine, Seoul, Republic of Korea.

The original Article has been corrected.

