# Supplementary material for: Genome-wide association study of metabolic dysfunction-associated fatty liver disease in a Korean population
Source: Sci Rep. 2024 Apr 29;14:9753. doi: 10.1038/s41598-024-60152-0 (PMC11056367; doi:10.1038/s41598-024-60152-0)
Supplement: Supplementary file 1 — Supplementary Tables. [file 41598_2024_60152_MOESM1_ESM.docx]

**Supplementary Table 1**. SNPs associated with metabolic dysfunction associated fatty liver disease: Conditional analysis with multiple lead SNPs on same chromosome.

|  | | | | | Discovery | | Validation | | Joint | |
| --- | --- | --- | --- | --- | --- | --- | --- | --- | --- | --- |
| SNP | Chr | Position | Nearest Genes | Risk Allele | OR (95% CI) | *P*-value | OR (95% CI) | *P*-value | OR (95% CI) | *P*-value |
| rs738409 | 22 | 44324727 | *PNPLA3* | G | 1.321  (1.186, 1.471) | 4.32E-07 | 1.230  (1.006, 1.503) | 0.043 | 1.298  (1.180, 1.427) | 7.21E-08 |
| rs3810622 | 22 | 44338134 | *PNPLA3* | T | 1.054  (0.944, 1.177) | 0.348 | 1.087  (0.890, 1.328) | 0.412 | 1.062  (0.965, 1.169) | 0.220 |

*P* values are calculated by binary logistic regression adjusted for age, sex, and five principal component scores assuming additive genetic model.

SNP, single nuclear polymorphism; CI, confidence interval; OR, odds ratio; Chr, chromosome

**Supplementary** **Table 2**. SNP associated with the subtype of metabolic dysfunction associated fatty liver disease: Conditional analysis with multiple lead SNPs.

|  | | | | | Discovery | | Validation | | Joint | |
| --- | --- | --- | --- | --- | --- | --- | --- | --- | --- | --- |
| SNP | Chr | Position | Nearest Genes | Risk Allele | OR (95% CI) | *P*-value | OR (95% CI) | *P*-value | OR (95% CI) | *P*-value |
| rs738409 | 22 | 44324727 | *PNPLA3* | G | 1.274  (1.148, 1.415) | 5.66E-06 | 1.205  (1.105, 1.438) | 0.063 | 1.257  (1.146, 1.379) | 1.15E-06 |
| rs3810622 | 22 | 44338134 | *PNPLA3* | T | 1.042  (0.936, 1.160) | 0.452 | 1.074  (0.883, 1.307) | 0.475 | 1.050  (0.956, 1.154) | 0.306 |

*P* values are calculated by ordinal logistic regression (proportional odds model) adjusted for age, sex, and five principal component scores assuming additive genetic model.

Genomic position is based on NCBI build 37.

SNP, single nuclear polymorphism; CI, confidence interval; OR, odds ratio; Chr, chromosome

**Supplementary Table 3**. The association between SNPs and subtype of metabolic dysfunction associated fatty liver disease: Conditional analysis with multiple lead SNPs.

| SNP | Risk Allele |  | Discovery | | Validation | | Joint | | |
| --- | --- | --- | --- | --- | --- | --- | --- | --- | --- |
| rs738409 | G | MAFLD subtype | OR (95% CI) | *P*-value | OR (95% CI) | *P*-value | OR (95% CI) | | *P*-value |
|  |  | No MAFLD | 1 (reference) |  | 1 (reference) |  | 1 (reference) |  | |
|  |  | Overweight/obese MAFLD | 1.436  (1.220, 1.691) | 1.41E-05 | 1.256  (0.913, 1.727) | 0.161 | 1.396  (1.207, 1.614) | 6.72E-06 | |
|  |  | MD-MAFLD | 1.268  (1.122, 1.433) | 1.37E-04 | 1.215  (0.967, 1.527) | 0.095 | 1.253  (1.125, 1.396) | 3.92E-05 | |
|  |  |  | Discovery | | Validation | | Joint | | |
| rs3810622 | T | MAFLD subtype | OR (95% CI) | *P*-value | OR (95% CI) | *P*-value | OR (95% CI) | *P*-value | |
|  |  | No MAFLD | 1 (reference) |  | 1 (reference) |  | 1 (reference) |  | |
|  |  | Overweight/obese MAFLD | 1.096  (0.925, 1.299) | 0.289 | 1.230  (0.889, 1.702) | 0.212 | 1.123  (0.966, 1.305) | 0.130 | |
|  |  | MD-MAFLD | 1.037  (0.915, 1.174) | 0.571 | 1.035  (0.825, 1.298) | 0.767 | 1.037  (0.930, 1.156) | 0.513 | |

*P* values are calculated by multinomial logistic regression adjusted for age, sex, and five principal component scores assuming additive genetic model.

MAFLD, metabolic dysfunction associated fatty liver disease; CI, confidence interval; OR, odds ratio; MD, metabolic dysfunction.
